# Identification of sex-specific genetic associations in response to opioid analgesics in a White, non-Hispanic cohort from Southeast Minnesota

**DOI:** 10.1038/s41397-022-00265-9

**Published:** 2022-01-31

**Authors:** Guilherme S. Lopes, Jaime L. Lopes, Suzette J. Bielinski, Sebastian M. Armasu, Ye Zhu, Dana C. Cavanaugh, Ann M. Moyer, Debra J. Jacobson, Liwei Wang, Ruoxiang Jiang, Jennifer L. St. Sauver, Nicholas B. Larson

**Affiliations:** 1 Premier, Inc, Charlotte, NC USA; 2 Cincinnati Children’s Hospital Medical Center, Cincinnati, OH USA; 3 Mayo Clinic, Rochester, MN USA; 4 NYU Langone Health Hospital, New York City, NY USA

**Keywords:** Risk factors, Genetic association study, Genetics research, Signs and symptoms

## Abstract

The study of sex-specific genetic associations with opioid response may improve the understanding of inter-individual variability in pain treatments. We investigated sex-specific associations between genetic variation and opioid response. We identified participants in the RIGHT Study prescribed codeine, tramadol, hydrocodone, and oxycodone between 01/01/2005 and 12/31/2017. Prescriptions were collapsed into codeine/tramadol and hydrocodone/oxycodone. Outcomes included poor pain control and adverse reactions within six weeks after prescription date. We performed gene-level and single-variant association analyses stratified by sex. We included 7169 non-Hispanic white participants and a total of 1940 common and low-frequency variants (MAF > 0.01). Common variants in *MACROD2* (rs76026520), *CYP1B1* (rs1056837, rs1056836), and *CYP2D6* (rs35742686) were associated with outcomes. At the gene level, *FAAH*, *SCN1A*, and *TYMS* had associations for men and women, and *NAT2*, *CYP3A4*, *CYP1A2*, and *SLC22A2* had associations for men only. Our findings highlight the importance of considering sex in association studies on opioid response.

## Introduction

Opioids are among the most commonly prescribed medications in the United States, with 51 opioid prescriptions per 100 persons filled in 2018 [1]. Despite the widespread use of opioids, there are important individual differences in opioid response in terms of pain relief, adverse reactions, and addiction [2–4]. Some of the variability is attributable to genetic factors, which are known to contribute to significant variability in pain perception and opioid metabolism [5, 6]. Genetic factors have explained about 30–76% of the inter-individual variability in opioid response in animal models [7]. To date, genetic association studies have reported over 150 genes associated with chronic pain conditions [5], some of which are also implicated in the risk of poor pain control and adverse reactions due to opioid analgesics [8]. For example, the cytochrome P450 2D6 enzyme is involved in prodrug conversion of opioids to their active metabolites. Specifically, genetic variation in *CYP2D6* directly affects CYP2D6 enzyme activity [9], which can predict a person’s likelihood to experience poor pain control and adverse reactions [10]. However, despite much progress in the field of individualized medicine, opioid-based treatments still have a high degree of inter-individual response variability attributable to their pharmacokinetic and pharmacodynamic properties [5]. Identifying novel genetic risk factors associated with opioid metabolism may help explain this variability and lead to more optimized treatment regimens.

In addition to genetic factors, response to opioid medications has been shown to vary by sex. For example, women are more likely to experience adverse reactions after opioid administration (e.g., nausea and vomiting) [11, 12]. Genetic loci associated with pain perception plays an important role in treatment effectiveness [5, 13], and some genetic loci associated with pain perception have also been demonstrated to be sex specific [14]. However, few pharmacogenomics (PGx) studies on pain perception and opioid metabolism have stratified results by sex. Among those studies, even fewer focused on specific outcomes of opioid medications, such as pain control and adverse reactions, with mixed results [15–17].

Together, these studies show that only a limited number of genetic variants have been identified as important in responses to opioid medications, and even fewer studies have examined the role of sex and genetic variation interactions. Identification of sex-specific genetic associations with opioid response may help clinicians to better individualize pain treatment, thereby optimizing effectiveness and reducing adverse reactions related to opioid use. To address these questions, we investigated sex-specific associations between genetic variation captured by a PGx sequencing panel and response to four commonly prescribed opioids in a large sample of individuals.

## Methods

### Study participants

Details of the RIGHT 10K Study have been previously reported [18–20]. Briefly, 10074 participants in the Mayo Clinic Biobank were invited and agreed to participate in a study of pre-emptive sequencing to understand the effect of variation in genes related to drug metabolism on drug outcomes. PGx sequencing results were deposited into the electronic health record (EHR) for use in clinical care. At the time of enrollment, participants in the RIGHT study provided written informed consent to allow their PGx genetic data and EHR data to be used for research in future studies. This study was approved by Institutional Review Boards (IRB) at the Mayo Clinic and Olmsted Medical Center, and was conducted in accordance with the Declaration of Helsinki.

### Opioid prescription

The Rochester Epidemiology Project (REP) is a medical records-linkage system connecting collaborating clinics, hospitals, and other medical facilities in Minnesota and Wisconsin, United States, and includes data of community members who have agreed to share their medical records for research [21]. The REP captures medical data from all participants in the RIGHT Study. We therefore utilized the REP research infrastructure to identify all participants in the RIGHT Study who were prescribed formulations containing codeine, tramadol, hydrocodone, or oxycodone between 1/1/2005 and 12/31/2017. We focused on these four opioids because they are commonly prescribed in the general population. We used RxNorm—a national database of normalized names for clinical drugs—to identify all codes for prescriptions that included one of the four opioids. We excluded formulations intended primarily to treat cough (e.g., guaifenesin/codeine). Participants prescribed one of the four opioids between 1/1/2005 and 12/31/2005 who also had an opioid prescription between 1/1/2004 and 12/31/2004 were excluded to focus on initiators of opioid use. Some formulations included nonsteroidal anti-inflammatory drugs (e.g., Ibuprofen/Oxycodone), which may contribute to increased pain relief beyond the effects of the opioid. Our study focused on formulations containing opioids. A complete list of formulations considered is shown in Supplemental Table 1.

### Outcomes

Opioid-related outcomes included a binary indication (yes/no) of poor pain control (e.g., clinical note indicating need to change dose or type of drug due to uncontrolled pain) and adverse reactions related to medication use (e.g., nausea, itching, constipation) attributed to the prescription within 6 weeks after each opioid prescription date. To identify potential opioid-related outcomes, EHRs were initially screened by natural language processing techniques using MedTagger [22]. Specifically, sentences including each opioid’s ingredient or trade names and keywords related to poor pain control or presence of adverse reactions due to opioid use were extracted from the clinical notes. All sentences were then reviewed by trained abstractors (JLS, DCC, and YZ), and classified as poor pain control (yes/no) or adverse reaction (yes/no). For example, the sentence “Patient has been taking Ultram but continues to complain of severe neck pain” was considered an indication of poor pain control associated with tramadol. This process is summarized in Supplemental Fig. 1. All records were reviewed blinded to the patients’ genetic information. Outcomes that could not be associated with a specific opioid prescription were not included (e.g., the sentence “feels nausea due to medication” written within 6 weeks of an opioid prescription does not specify whether this adverse reaction is due to the prescribed opioid or to another medication).

### PGx sequencing

PGx sequencing was performed by the Clinical Laboratory Improvement Amendments-certified and College of American Pathologists-accredited Baylor College of Medicine’s Human Genome Sequencing Center Clinical Laboratory using the Pharmacogenomics Research Network (PGRN)-Seq v3 targeted capture sequencing panel. Details of the PGx sequencing in the RIGHT Study have been previously reported [18–20]. To potentially improve downstream quality control analyses that traditionally rely on genome-wide data, we additionally performed genome-wide genotyping of off-capture regions. Additional variant level annotation was assigned to all called variants using BioR [23], including output from Clinical Annotation of Variants (CAVA) [24].

### Quality control

Variants were excluded from analysis if the variant call rate among all sequenced participants was <0.95. Despite the limited amount of genetic variation captured by the PGRN-Seq v3 assay, we attempted preliminary evaluation of population substructure and relatedness using both on- and off-capture genotype calls. Population structure was evaluated via LASER using Fast and Robust Ancestry Coordinate Estimation and the Human Genome Diversity Project reference panels, while relatedness was assessed using PREST. Results were largely consistent with self-reported race/ethnicity (Supplemental Fig. 2), and thus we applied the latter as the basis for subsetting to an ancestrally homogenous subgroup of RIGHT participants. Results from PREST indicated evidence of substantial relatedness present, although identity-by-descent estimates were too noisy to confidently resolve genetic relationships (Supplemental Fig. 3). To fully leverage all available data, we considered mixed-model genetic association analysis approaches that can accommodate cryptic relatedness and latent population substructure using an estimated genetic relatedness matrix (GRM). For variants among self-reported non-Hispanic whites, we estimated the minor allele frequency (MAF) and conducted Hardy–Weinberg equilibrium (HWE) for variants with empirical MAF > 0.05. Given that the presence of cryptic relatedness can lead to HWE test Type I error inflation, we considered significant deviation from HWE at a conservative *p* value < 1 × 10^−5^ and excluded such variants from further single-variant analysis.

### Statistical analyses

Patient characteristics were summarized using counts and percentages for categorical variables, and median and quartiles for continuous variables. Sex was retrieved from the EHR. Age was calculated for each date of opioid’s prescription.

To improve sample size in analysis, we performed analyses by related drug groups based on participants who were prescribed codeine and/or tramadol or hydrocodone and/or oxycodone. These groupings follow previous research [16, 25] and are based on drug similarities in pharmacodynamic profiles. For example, hydrocodone and oxycodone are phenanthrenes, and patients report similar pain relief and adverse reactions to hydrocodone and oxycodone, including nausea, vomiting, itching, and drowsiness [26]. In contrast, codeine, which contains the 6-hydroxyl groups, is associated with more nausea and hallucinations than hydrocodone and oxycodone, which do not have the 6-hydroxyl groups [27]. In addition, the difference in affinity for the opioid receptor between parent drug and active metabolite is not as large for hydrocodone and oxycodone compared to tramadol and codeine. Under this analysis design, participants could be included twice in an analysis if they were prescribed drugs from different groupings.

For each single-SNP association analysis, autosomal variants with an empirical MAF > 0.01 among analyzed participants were considered for testing, and analyses were performed both combined across and stratified by sex. To account for residual population stratification and cryptic relatedness, we applied generalized linear mixed-effects (GLMM) modeling using the R package GMMAT [28]. GMMAT applies an estimated GRM to account for genetic similarity across study participants. A sparse GRM was generated such that entries < 0.125 were truncated to zero. We also allowed for a random intercept to account for multiple observations per participant with multiple drug exposures in a given drug group. A multivariable logistic GLMM was used to model associations between each variant in the risk of poor pain control or adverse reactions to each group of opioids. Testing was performed using the GMMAT score test under an additive genetic model while additionally adjusting for age at prescription, sex, and prescribed drug (e.g., codeine [yes/no] and tramadol [yes/no] for the codeine/tramadol group). The direction of effect was determined based on the sign of the score test statistic. Variant associations were declared to be statistically significant at a false discovery rate (FDR) threshold of < 0.05 using the Benjamini–Hochberg approach, which was also used to compute FDR-adjusted *p* values (*q* values). FDR control was conducted stratified by each analysis (2 drug groups × 3 cohorts × 2 outcomes = 12 total analyses). Suggestive variant associations were also highlighted at a *p* value < 0.001.

Additionally, we conducted a gene-level analysis to investigate the aggregate effects of common and rare variants for all directly sequenced genes. Specifically, we applied the efficient hybrid test SMMAT-E implemented in GMMAT under the same models described above for single-variant association analysis, restricted to variants located within the respective gene boundaries. Default MAF-based beta weights were applied to place more weight on rarer variation. Given the relatively small number of genes and likely weak genetic correlation across genes, we adopted a simple Bonferroni-corrected alpha of 0.05 to define statistical significance of gene-based testing, where the correction factors were based on total number of genes tested and sex strata (77 genes by 2 sexes). We conducted analyses including a filtered subset of identified variants based on CAVA impact values of “moderate” or “high”, as these single nucleotide polymorphisms (SNPs) are more likely to affect protein function. To aid in the interpretation of gene-level results, we additionally examined the direction of effect (i.e., risk or protective) from the SMMAT burden test output.

## Results

Among 10077 RIGHT participants, 2393 (24%) participants were excluded because they were not prescribed any opioids or were not initiators of opioid use within the study date range (1/1/2005–12/31/2017). An additional 515 (6.7%) participants were excluded due to self-reported non-white race and/or Hispanic ethnicity. The final sample included 7169 participants, of which 3636 (51%) were prescribed codeine/tramadol, and 6649 (93%) were prescribed hydrocodone/oxycodone. Additionally, 4284 participants were prescribed two or more opioids; 1069 (25%) were prescribed opioids within 6 weeks of each other, and 784 (11%) were prescribed opioids from different groups (e.g., codeine and oxycodone) within 6 weeks of each other. Participant characteristics are shown in Table 1.

**Table 1:** Patient characteristics by sex among patients prescribed codeine/tramadol (*n* = 3636) and hydrocodone/oxycodone (*n* = 6649).

Characteristic	Codeine/Tramadol			Hydrocodone/Oxycodone			Total
	Men	Women	Total	Men	Women	Total	
Total of participants	1382	2254	3636	2571	4078	6649	7169
Age at first prescription^a^							
Median	67	60	63	64	56	60	59
Q1–Q3	59–72	48–69	52–70	56–70	43–65	47–68	47–67
Unknown	0	0	0	0	0	0	0
Body Mass Index							
Median	29	28	29	29	28	28	28
Q1–Q3	27–33	24–33	25–33	26–33	24–33	25–33	25–33
Unknown	0	0	0	0	1	1	1
Poor pain control, *n* (%)							
Yes	72 (5.2)	117 (5.2)	189 (5.2)	89 (3.5)	151 (3.7)	240 (3.6)	409 (5.7)
No	1310 (95)	2137 (95)	3447 (95)	2482 (97)	3927 (96)	6409 (96)	6760 (94)
Unknown	0	0	0	0	0	0	0
Adverse reactions, *n* (%)							
Yes	56 (4.1)	154 (6.8)	210 (5.8)	77 (3)	163 (4)	240 (3.6)	432 (6)
No	1326 (96)	2100 (93)	3426 (94)	2494 (97)	3915 (96)	6409 (96)	6737 (94)
Unknown	0	0	0	0	0	0	0

Note: Percentages are by column.

^a^Age at first prescription within each opioid group (e.g., for codeine/tramadol, age was calculated at the earliest prescription between codeine and tramadol).

### Association of variants with opioid response

A total of 1940 common and low-frequency SNPs (MAF > 0.01) were included in at least one analysis. Supplemental Fig. 4 displays the Manhattan plots representing pooled and sex-stratified analyses for all variants. Among participants prescribed hydrocodone/oxycodone (*n* = 6649), one intronic SNP was associated with a decreased risk of poor pain control in the pooled sample (rs76026520, *MACROD2*: *p* value = 2.1 × 10^−5^, *q* value = 0.0397). Among participants prescribed codeine/tramadol (*n* = 3636), two SNPs, one synonymous and one nonsynonymous, were associated with an increased risk of poor pain control in men (rs1056837, *CYP1B1*: *p* value = 7.8 × 10^−6^, *q* value = 0.0075; rs1056836, *CYP1B1*: *p* value = 8 × 10^−6^, *q* value = 0.0075, respectively). Among variants identified in *CYP2D6*, rs35742686 demonstrated the strongest association in any analysis, with the alternate allele dosage associated with decreased risk of adverse reactions to hydrocodone/oxycodone in the pooled sample (*p* value = 7.5 × 10^−5^, *q* value = 0.1436). However, this association did not meet our statistical significance criterion. Table 2 summarizes the characteristics of these SNPs. A complete list of common variants associated with opioid response at the suggestive threshold of *p* value < 0.001 is summarized in Supplementary Table 2.

**Table 2:** Highlighted associations between common variants and opioid response.

Opioids	Outcome	Sex	rsID	Gene	Major	Minor	MAF^a^	Effect	Direction	*P* value	*Q* value	CAVA impact
Codeine/Tramadol	Poor pain control	Male	rs1056837	*CYP1B1*	G	A	0.433	Synonymous	Risk	7.8 × 10^−6^	0.0076	Low
			rs1056836	*CYP1B1*	G	C	0.432	Missense	Risk	8.0 × 10^−^^6^	0.0076	Moderate
Hydrocodone/Oxycodone	Poor pain control	All	rs76026520	*MACROD2*	A	G	0.094	Intronic	Protective	2.1 × 10^−^^5^	0.0397	Low
	Adverse events	All	rs35742686	*CYP2D6*	CT	C	0.019	Nonsense	Protective	7.6 × 10^−^^5^	0.1436	High

^a^MAF empirical to the current dataset.

### Association of genes with opioid response

A total of 77 genes were included in at least one gene-level analysis in our study, and a *p* value ≤ 0.05/77 × 2 (i.e. 3.2 × 10^−4^; Bonferroni correction) was considered statistically significant among all tests. From these analyses, a total of seven genes were significantly associated with different opioid outcomes. Table 3 summarizes the genes associated with opioid response with the functional impact variant filter. The *FAAH* gene was associated with an increased risk of adverse reactions to codeine/tramadol in the pooled sample (*p* value = 2.0 × 10^−7^), and separately for men (*p* value = 2.1 × 10^−6^) and women (*p* value = 1.0 × 10^−4^). The *FAAH* gene was also associated with an increased risk of adverse reactions to hydrocodone/oxycodone in the pooled sample (*p* value = 7.6 × 10^−5^), and this association was primarily driven by men (*p* value = 1.4 × 10^−10^). In addition, the *TYMS* gene was associated with an increased risk of poor pain control to codeine/tramadol in the pooled sample (*p* value = 3.5 × 10^−5^), and this association was primarily driven by men (*p* value = 1.1 × 10^−6^). Finally, the *SCN1A* gene was associated with an increased risk of adverse reactions to codeine/tramadol in the pooled sample (*p* value = 2.0 × 10^−4^), with the strongest association observed in women (*p* value = 5.0 × 10^−4^).

**Table 3:** Genes associated with opioid response at an adjusted *p* value ≤ 0.05.

Opioids	Outcome	Gene	All			Men			Women		
			Burden^c^	No. of variants^a^	*P* value^b^	Burden^c^	No. of variants^a^	P value ^b^	Burden^c^	No. of variants^a^	*P* value^b^
Codeine/Tramadol	Adverse reactions	*FAAH*	**Risk**	**24**	**1.7 × 10**^**−7**^	**Risk**	**12**	**2.1 × 10**^**−6**^	**Risk**	**18**	**0.0001**
		*SCN1A*	**Risk**	**42**	**0.0002**	Risk	23	0.47	Risk	26	0.0005
		*NAT2*	Risk	27	0.0031	**Risk**	**15**	**0.0002**	Risk	18	0.22
		*CYP3A4*	Risk	33	0.013	**Risk**	**19**	**0.0001**	Risk	21	0.30
	Poor pain control	*TYMS*	**Risk**	**8**	**3.5 × 10**^**−5**^	**Risk**	**6**	**1.1 × 10**^**−6**^	Risk	5	0.30
		*CYP1A2*	Risk	29	0.038	**Risk**	**21**	**0.0001**	Protective	16	0.83
Hydrocodone/Oxycodone	Adverse reactions	*FAAH*	**Risk**	**40**	**7.6 × 10**^**−5**^	**Risk**	**18**	**1.4 × 10**^**−10**^	Risk	29	0.64
	Poor pain control	*SLC22A2*	Risk	53	0.0017	**Risk**	**31**	**9.3 × 10**^**−6**^	Risk	33	0.61

Bold cells indicate results are statistically significant.

^a^Number of variants based on a functional impact filter defined as assigned CAVA impact tier of “moderate” or “high”.

^b^*P* values ≤ 3.3 × 10^−4^ (Bonferroni correction) were considered statistically significant.

^c^Defined by the sign of the burden test statistic, where positive values indicate genetic burden associated with increased risk.

Some associations were significant only among men. In particular, the *NAT2* and *CYP3A4* genes were associated with increased risk of adverse reactions to codeine/tramadol (*p* value = 2.0 × 10^−4^ and *p* value = 1.0 × 10^−4^, respectively), and the *CYP1A2* gene was associated with increased risk of poor pain control to codeine/tramadol (*p* value = 1.0 × 10^−4^). Finally, the *SLC22A2* gene was also associated with increased risk of poor pain control to hydrocodone/oxycodone in men (*p* value = 9.3 × 10^−6^).

## Discussion

In this study, we investigated the effects of genetic variation on with adverse reactions and poor pain control to four commonly prescribed opioid medications. Common variants in *MACROD2*, *CYP1B1*, and *CYP2D6* were associated with these outcomes. At the gene level, *FAAH*, *SCN1A*, and *TYMS* were also associated with these outcomes. Finally, we stratified our results by sex and found that *NAT2*, *CYP3A4*, *CYP1A2*, and *SLC22A2* were associated with outcomes in men (but not in women).

### Common variants and opioid response

*CYP2D6* encodes an important metabolic enzyme that converts prodrugs—such as the opioids—to their active metabolites. Genetic polymorphisms in this gene impact enzyme activity, which can be classified as poor, intermediate, normal, or ultra-rapid based on the effect of variants on the resulting protein structure and quantity. Among all *CYP2D6* single-variant associations we examined, rs35742686 (p.R259Gfs*2, MAF = 0.018) was suggestively associated with a decreased risk of adverse reactions to hydrocodone/oxycodone in our pooled cohort. This variant defines the CYP2D6*3 allele, which is inactive. Loss of function variants lead to reduced enzymatic activity and poor metabolism status [9]. Individuals with a *CYP2D6* poor metabolizer phenotype—such as homozygotes of the CYP2D6*3 allele—have more limited conversion of prodrugs into their active metabolites, and therefore are less likely to experience adverse reactions relative to individuals with a *CYP2D6* normal metabolizer phenotype [10, 16]. The marginal nature of the association in our study may be due to the relatively low minor allele frequency for this variant (MAF = 0.019) and therefore decreased power. Other *CYP2D6* single-variant associations examined included rs3892097 (c.506−1G > A, MAF = 0.20), which defines the inactive CYP2D6*4 allele. We did not find significant associations for this variant across outcomes and cohorts (all *p* values ≥ 0.05). One possible explanation is the complex genetics of *CYP2D6* metabolizer phenotypes, including copy-number states [29, 30], for which simple additive genetic models may inadequately capture genetic relationships with risk. Future research involving more complex copy-number and haplotype-based approaches are warranted.

We identified two variants associated with increased risk of poor pain control to codeine/tramadol among men in the *CYP1B1* gene (rs1056837 and rs1056836). *CYP1B1* encodes a cytochrome P450 monooxygenase involved in the metabolism of various endogenous substrates, including fatty acids, steroid hormones, and vitamins [31–35]. Similar to *CYP2D6*, *CYP1B1* is involved in phase 1 drug metabolism. However, *CYP1B1* is a relatively recently identified member of the CYP1 gene family and associations between this gene and opioid response are not yet established. Finally, one variant in the *MACROD2* gene (rs76026520) was associated with increased risk of poor pain control to hydrocodone/oxycodone in the pooled sample. The *MACROD2* gene has been associated with estrogen-independent growth and tamoxifen resistance in breast cancer patients [36], with obesity in the Korean population [37], and with autism spectrum disorder in the general population [38]. It is unclear whether MACROD2 plays a role in response to opioids.

### Association of genes with opioid response

Variation in the *FAAH* gene has been previously associated with both pain perception and adverse reactions with use of morphine derivatives [15, 17]. Consistent with the literature, *FAAH* variation was associated with an increased risk of adverse reactions to both opioid groups. For codeine/tramadol, we observed this association in both men and women. For hydrocodone/oxycodone, this association was in the pooled sample but driven primarily by men. *FAAH* (fatty acid amide hydrolase) encodes a serine hydrolase responsible for the degradation of fatty acid amide family of signaling lipids. FAAH targets include endocannabinoid ligands which regulate pain, inflammation, and several cognitive and emotional states (e.g., anxiety, depression) [39]. FAAH inhibitors have been shown to augment endocannabinoid signaling, thus reducing inflammation and producing pain relief comparable to tramadol [40]. Therefore, genetic variants leading to reduced FAAH protein function would be expected to exert a similar effect.

*FAAH* variant rs324420 (p.Pro129Thr, MAF = 0.20) is a well-characterized missense variant shown to lead to protein instability and decreased cellular *FAAH* expression [41]. This variant has been associated with increased risk of refractory post-operative nausea/vomiting, opioid-related respiratory depression [15], and increased risk for substance abuse [42]. We uncovered a significant genetic burden of the gene *FAAH* associated with an increased risk of adverse reactions to codeine/tramadol (in men and women separately), and to hydrocodone/oxycodone (in the pooled sample, but driven primarily by men). However, we did not observe any association between opioid outcomes and individual *FAAH* SNPs, including variant rs324420. This may be due to the fact that variants with deleterious impact are more likely to be rare. For this reason, studies with increased sensitivity of biologically relevant results often perform both variant- and gene-level analyses.

The *SCN1A* gene was associated with an increased risk of adverse reactions to codeine/tramadol, and this association was driven primarily by women. The *SCN1A* gene is mainly expressed in cerebral tissue, and mutant SCN1A channels are highly heterogeneous. *SCN1A* has been associated with an increased risk of childhood seizures [43] and sudden infant death syndrome [44]. Future research may investigate associations between SCN1A phenotypes and opioid response in men and women. In addition, the *TYMS* gene was associated with an increased risk of poor pain control to codeine/tramadol, and this association was driven primarily by men. *TYMS* encodes for thymidylate synthase. Variants in the *TYMS* gene, which lead to increased thymidylate synthase expression, have been associated with poor response to methotrexate, an immunosuppressive drug [45, 46]. It is unclear whether *TYMS* plays a role in the response to other drugs.

### Sex-specific associations of genes with opioid response

We found that the *NAT2* and *CYP3A4* genes were associated with increased risk of adverse reactions to codeine/tramadol among men, but not women. *NAT2* encodes an N-acetyltransferase that activates and deactivates various exogenous drugs and carcinogens. Polymorphisms in *NAT2* can lead to rapid, intermediate, and slow metabolizer phenotypes and are associated with drug toxicity [47]. The *CYP3A4* gene is a member of the cytochrome P450 gene family, encoding for key enzymes involved in drug metabolism. Our finding is consistent with previous research documenting the role of *CYP3A4* in the pharmacodynamics of opioids, including fentanyl, tramadol, and oxycodone [48, 49]. Our sex-specific finding is also consistent with research indicating that sex is an important factor of *CYP3A4* expression [50].

In addition, *CYP1A2* was associated with increased risk of poor pain control to codeine/tramadol among men, but not women. CYP1A2 activity has been demonstrated to differ by sex [51], with estradiol being a contributing factor for this sex difference [52]. Finally, the *SLC22A2* gene was associated with increased risk of poor pain control to hydrocodone/oxycodone among men, but not women. The *SLC22A2* gene encodes for an important drug uptake transporter, and inhibition of SLC22A2 has been documented to affect morphine metabolism in mouse models [53]. However, more studies are needed to examine whether sex modifies the associations between *SLC22A2* metabolizer phenotypes and opioid response.

Previous research suggested that interactions between sex and genetics on pain perception and opioid metabolism may be due to sex differences in the functioning of receptors (e.g., μ-, δ-opioid) and enzymes important to opioid pathways [54]. We have previously observed sex-specific associations between response to codeine and tramadol and CYP2D6 phenotypes [16]. However, Sadhasivam et al. [15]. documented SNPs associated with adverse reactions due to morphine administration in children, but the associations did not differ by sex. In addition, baseline pain sensitivity may affect opioid-based therapy effectiveness. Among candidate gene association studies, Kim et al [17]. reported variation in the *FAAH* gene associated with cold pain sensitivity in European American men, but not in women.

PGx studies on opioids reporting sex-specific results are still scarce, despite NIH guidelines for clinical research stating the importance of sex as a biological variable to be factored in research design and statistical analysis [55]. The lack of sex-specific reports in PGx studies could lead to inappropriately broad assumptions on the effectiveness of pain therapies. Changes in clinical care based on these assumptions could negatively impact therapeutic response. Our findings add to a growing body of evidence indicating sex-specific genetic associations with opioid response.

## Strengths, limitations, and conclusion

The strengths of this study include a large sample size and an ability to extract information directly from the EHR. In addition, we had an extensive PGx sequencing data and complete follow-up information after opioid prescription for all patients. However, there are several limitations. Patient outcomes collected retrospectively from EHRs are limited to those reported to a health care provider. Active, real-time follow-up of persons prescribed opioid analgesics may be needed to disentangle drug response from other factors influencing individuals’ willingness to report outcomes to health care providers. In addition, 784 (11%) of all prescribed patients were prescribed opioids from different groups (e.g., codeine and oxycodone) within 6 weeks of each other. Although an effort was made to associate outcomes to their corresponding opioids, some of the reported outcomes may have been due to other prescriptions or health events that occurred within 6 weeks of the prescription and were erroneously attributed to the opioid medication. Future research may be powered specifically to investigate PGx-wide associations with response to each opioid separately. Some patients may have not adhered to their opioid prescription, and we could not investigate the effects of use frequency or dosage on pain control and risk of adverse reactions. Type of adverse reactions and diagnosis associated with opioid prescription were not available for this cohort. Future research may investigate sex-specific associations between genetic variation and opioid response for specific adverse reactions or in cohorts of patients with specific diagnosis (e.g., oncological patients). The RIGHT protocol included a targeted panel of genes, and therefore our analysis did not permit investigation of genetic variation outside the capture design. Genome-wide association studies may reveal novel SNPs implicated in opioid response. In addition, many PGx genes exert phenotype via gene duplication and may be better uncovered using expression studies such as RNAseq. Finally, our cohort was from a non-Hispanic white population, and may not be generalizable to other populations.

In summary, we investigated sex-specific associations of PGx genes and their variants with response to commonly prescribed opioids in a large sample of individuals. We replicated previous findings that polymorphisms in some genes (e.g., *CYP2D6*, *CYP3A4*, *CYP1A2*) are related to opioid outcomes. We also identified novel genes for future studies of opioid response (e.g., *TYMS*, *SLC22A2*). Finally, our data suggest that there may be significant sex-based differences in the effects of these genes on opioid responses. Our findings highlight the importance of considering sex as a biological variable in the investigation of genetic associations with response to opioid analgesics.

## Supplementary information

Legends for Supplemental Tables and Supplemental Figures

Supplemental Table 1. Ingredients and RxNorm codes of opioid medications included in the study

Supplemental Table 2. Common variants associated with opioid response at a p-value < 0.001

Supplemental Figure 1 – Adverse reactions and poor pain control related to opioid use extracted from electronic health records

Supplemental Figure 2 – Leading principal components from TRACE with RIGHT participants projected onto reference samples from HGDP

Supplemental Figure 3 – IBD estimates for relatedness checks in RIGHT using PREST

Supplemental Figure 4 – Manhattan plot of PGx variant associations with opioid response by sex

## Data Availability

For data sharing requests, please contact SJB at Bielinski.Suzette@mayo.edu.

## Code availability

All analyses were conducted using R version 3.6.2. Codes available upon request.
