# Supplementary material for: Identification of sex-specific genetic associations in response to opioid analgesics in a White, non-Hispanic cohort from Southeast Minnesota
Source: Pharmacogenomics J. 2022 Jan 31;22(2):117–23. doi: 10.1038/s41397-022-00265-9 (PMC8975736; doi:10.1038/s41397-022-00265-9)
Supplement: Supplementary file 1 — Legends for Supplemental Tables and Supplemental Figures [file 41397_2022_265_MOESM1_ESM.docx]

**Supplemental table legends**

**Supplemental Table 1.** Ingredients and RxNorm codes of opioid medications included in the study

**Supplemental Table 2.** Common variants associated with opioid response at a p-value < 0.001

**Supplemental figure legends**

**Supplemental Figure 1** – Adverse reactions and poor pain control related to opioid use extracted from electronic health records

**Supplemental Figure 2** – Leading principal components from TRACE with RIGHT participants projected onto reference samples from HGDP (colored)

**Supplemental Figure 3** – IBD estimates for relatedness checks in RIGHT using PREST

**Supplemental Figure 4** – Manhattan plot of PGx variant associations with opioid response by sex; Note: Blue line indicates p-value < 0.001
