# Supplementary material for: Identification of sex-specific genetic associations in response to opioid analgesics in a White, non-Hispanic cohort from Southeast Minnesota
Source: Pharmacogenomics J. 2022 Jan 31;22(2):117–23. doi: 10.1038/s41397-022-00265-9 (PMC8975736; doi:10.1038/s41397-022-00265-9)
Supplement: Supplementary file 2 — Supplemental Table 1. Ingredients and RxNorm codes of opioid medications included in the study [file 41397_2022_265_MOESM2_ESM.docx]

**Supplemental Table 1.** Ingredients and RxNorm codes of opioid medications included in the study

| **Opioid** | **RxNorm code** | **Ingredient name** |
| --- | --- | --- |
| Codeine | 821601 | Acetaminophen/Aspirin/Caffeine/Codeine |
| Codeine | 689552 | Acetaminophen/Aspirin/Caffeine/Codeine/salicylamide |
| Codeine | 689555 | Acetaminophen/Aspirin/Codeine |
| Codeine | 689561 | Acetaminophen/butalbital/Caffeine/Codeine |
| Codeine | 689563 | Acetaminophen/butalbital/Codeine |
| Codeine | 814657 | Acetaminophen/Caffeine/Codeine |
| Codeine | 817430 | Acetaminophen/Caffeine/Codeine/Meprobamate |
| Codeine | 689568 | Acetaminophen/Caffeine/Codeine/salicylamide |
| Codeine | 817579 | Acetaminophen/Codeine |
| Codeine | 817356 | Acetaminophen/Codeine/Ibuprofen |
| Codeine | 1007238 | Aconite/Codeine/Erysimum preparation |
| Codeine | 690996 | Aluminum Hydroxide/Aspirin/Codeine/Magnesium Hydroxide |
| Codeine | 214237 | anhydrous calcium iodide/Codeine |
| Codeine | 214160 | Aspirin/butalbital/Caffeine/Codeine |
| Codeine | 689511 | Aspirin/Caffeine/Codeine |
| Codeine | 689522 | Aspirin/Carisoprodol/Codeine |
| Codeine | 135095 | Aspirin/Codeine |
| Codeine | 1008493 | Benzoate/Codeine |
| Codeine | 1008060 | Butalbital/Caffeine/Codeine |
| Codeine | 691032 | Calcium iodide/Codeine |
| Codeine | 2670 | Codeine |
| Codeine | 1008110 | Codeine/Diclofenac |
| Codeine | 1008954 | Codeine/Erysimum preparation |
| Codeine | 1007477 | Codeine/Ethylmorphine |
| Codeine | 710303 | Codeine/Ibuprofen |
| Codeine | 214443 | Codeine/iodinated glycerol |
| Codeine | 729517 | Codeine/Kaolin |
| Codeine | 690089 | Codeine/Papaverine |
| Codeine | 1007293 | Codeine/Potassium |
| Codeine | 690096 | Codeine/potassium citrate |
| Codeine | 1007204 | Codeine/propyphenazone |
| Codeine | 690101 | Codeine/Pyrilamine |
| Codeine | 689569 | Acetaminophen/Caffeine/dihydrocodeine |
| Codeine | 151196 | Acetaminophen/dihydrocodeine |
| Codeine | 689783 | Acetaminophen/dihydrocodeine/salicylamide |
| Codeine | 689512 | Aspirin/Caffeine/dihydrocodeine |
| Codeine | 23088 | Dihydrocodeine |
| Oxycodone | 214183 | Acetaminophen/Oxycodone |
| Oxycodone | 214256 | Aspirin/Oxycodone |
| Oxycodone | 484259 | Ibuprofen/Oxycodone |
| Oxycodone | 1545902 | Naloxone/Oxycodone |
| Oxycodone | 7804 | Oxycodone |
| Hydrocodone | 689553 | Acetaminophen/Aspirin/Caffeine/Hydrocodone |
| Hydrocodone | 689562 | Acetaminophen/butalbital/Caffeine/Hydrocodone |
| Hydrocodone | 214182 | Acetaminophen/Hydrocodone |
| Hydrocodone | 689515 | Aspirin/Caffeine/Hydrocodone |
| Hydrocodone | 214253 | Aspirin/Hydrocodone |
| Hydrocodone | 5489 | Hydrocodone |
| Hydrocodone | 214627 | Hydrocodone/Ibuprofen |
| Tramadol | 352362 | Acetaminophen/Tramadol |
| Tramadol | 10689 | Tramadol |
