# Supplementary material for: Identification of sex-specific genetic associations in response to opioid analgesics in a White, non-Hispanic cohort from Southeast Minnesota
Source: Pharmacogenomics J. 2022 Jan 31;22(2):117–23. doi: 10.1038/s41397-022-00265-9 (PMC8975736; doi:10.1038/s41397-022-00265-9)
Supplement: Supplementary file 3 — Supplemental Table 2. Common variants associated with opioid response at a p-value < 0.001 [file 41397_2022_265_MOESM3_ESM.docx]

**Supplemental Table 2.** Common variants associated with opioid response at a p-value < 0.001

| **Opioids** | **Outcome** | **Sex** | **rsID** | **Gene** | **Major** | **Minor** | **MAF*** | **Effect** | **Direction** | **P-value** | **Q-value** | **CAVA impact** |
| --- | --- | --- | --- | --- | --- | --- | --- | --- | --- | --- | --- | --- |
| **Codeine/Tramadol** | **Adverse reactions** | **Female** | rs7877 | *FMO1* | C | T | 0.275 | 3' UTR | Protective | 9.5 × 10^-5^ | 0.1807 | LOW |
|  |  |  | rs3755740 | *--* | G | A | 0.272 | Intronic | Risk | 0.0009 | 0.3191 | LOW |
|  |  |  | rs2259816 | *HNF1A* | G | T | 0.358 | 3' UTR | Risk | 0.0009 | 0.3191 | LOW |
|  |  |  | rs3755739 | *--* | G | A | 0.275 | Intronic | Risk | 0.0010 | 0.3191 | LOW |
|  |  | **Male** | rs55750514 | *CYP2D8P* | G | C | 0.073 | Intronic | Protective | 0.0005 | 0.7610 | LOW |
|  |  |  | rs2835266 | *CBR1* | G | A | 0.057 | Missense | Protective | 0.0008 | 0.7610 | LOW |
|  |  | **All** | rs3755906 | *IGFBP7* | A | T | 0.384 | Splice | Protective | 0.0003 | 0.3567 | LOW |
|  |  |  | rs3755740 | *--* | G | A | 0.272 | Intronic | Risk | 0.0008 | 0.3567 | LOW |
|  |  |  | rs3755739 | *--* | G | A | 0.274 | Intronic | Risk | 0.0008 | 0.3567 | LOW |
|  |  |  | rs4683739 | *--* | T | A | 0.248 | Intronic | Risk | 0.0008 | 0.3567 | LOW |
|  |  |  | rs2280083 | *CHST2* | G | A | 0.247 | 5' UTR | Risk | 0.0009 | 0.3567 | LOW |
|  | **Poor pain control** | **Male** | rs1056837 | *CYP1B1* | G | A | 0.433 | Synonymous | Risk | 7.8 × 10^-6^ | 0.0076 | LOW |
|  |  |  | rs1056836 | *CYP1B1* | G | C | 0.432 | Missense | Risk | 8.0 × 10^-6^ | 0.0076 | MODERATE |
|  |  |  | rs1046428 | *GSTZ1* | C | T | 0.197 | Missense | Protective | 0.0005 | 0.3004 | MODERATE |
|  |  | **All** | rs41545022 | *HLA-A* | C | G | 0.055 | 3' UTR | Risk | 0.0009 | 0.9199 | LOW |
| **Hydrocodone/Oxycodone** | **Adverse reactions** | **Female** | rs7785206 | *CROT* | G | C | 0.071 | Missense | Protective | 0.0002 | 0.2468 | MODERATE |
|  |  |  | rs2230028 | *ABCB4* | T | C | 0.073 | Missense | Protective | 0.0003 | 0.2468 | MODERATE |
|  |  | **Male** | rs2279341 | *CYP2B6* | G | C | 0.058 | Synonymous | Protective | 0.0002 | 0.2273 | LOW |
|  |  |  | rs8192709 | *CYP2B6* | C | T | 0.059 | Missense | Protective | 0.0002 | 0.2273 | MODERATE |
|  |  |  | rs2180314 | *GSTA2* | G | C | 0.401 | Missense | Risk | 0.0007 | 0.4327 | MODERATE |
|  |  | **All** | rs7785206 | *CROT* | G | C | 0.070 | Missense | Protective | 0.0007 | 0.4717 | MODERATE |
|  |  |  | rs6785049 | *NR1I2* | A | G | 0.387 | Intronic | Risk | 0.0007 | 0.4717 | LOW |
|  |  |  | rs35742686 | *CYP2D6* | CT | C | 0.019 | Nonsense | Protective | 7.6 × 10^-5^ | 0.1436 | HIGH |
|  | **Poor pain control** | **Female** | rs6729738 | *AOX3P* | T | C | 0.458 | Intronic | Protective | 0.0010 | 0.8032 | LOW |
|  |  |  | rs76026520 | *MACROD2* | A | G | 0.095 | Intronic | Protective | 0.0002 | 0.2899 | LOW |
|  |  | **Male** | rs4377447 | *CHST13/C3orF22* | G | T | 0.011 | Intronic | Protective | 0.0001 | 0.1319 | LOW |
|  |  |  | rs12721623 | *CYP3A4* | A | C | 0.017 | Intronic | Protective | 0.0005 | 0.4432 | LOW |
|  |  |  | rs77667132 | *CHST13/C3orF22* | C | T | 0.019 | Intronic | Protective | 0.0008 | 0.4432 | LOW |
|  |  |  | rs4993392 | *--* | C | T | 0.025 | Intronic | Protective | 0.0009 | 0.4432 | LOW |
|  |  | **All** | rs76026520 | *MACROD2* | A | G | 0.094 | Intronic | Protective | 2.1 × 10^-5^ | 0.0397 | LOW |

*MAF empirical to the current dataset

Note: Highlighted associations indicate SNPs with q-value < 0.05 and a SNP in the CYP2D6 gene.
