# Supplementary figures and images for: Identification of sex-specific genetic associations in response to opioid analgesics in a White, non-Hispanic cohort from Southeast Minnesota
Source: Pharmacogenomics J. 2022 Jan 31;22(2):117–23. doi: 10.1038/s41397-022-00265-9 (PMC8975736; doi:10.1038/s41397-022-00265-9)

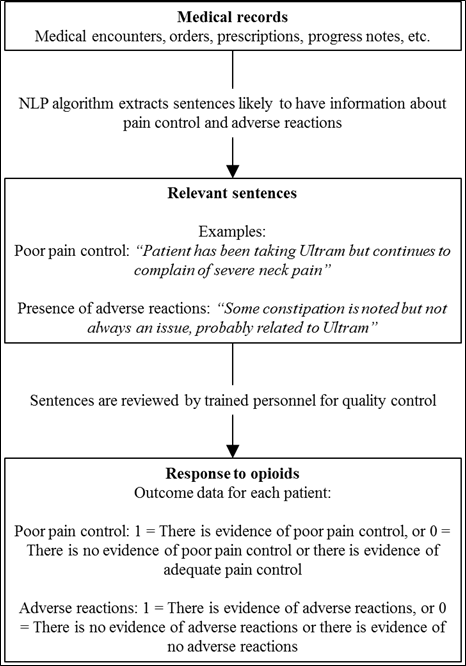

Supplement: Supplementary file 4 — Supplemental Figure 1 – Adverse reactions and poor pain control related to opioid use extracted from electronic health records [file 41397_2022_265_MOESM4_ESM.png]

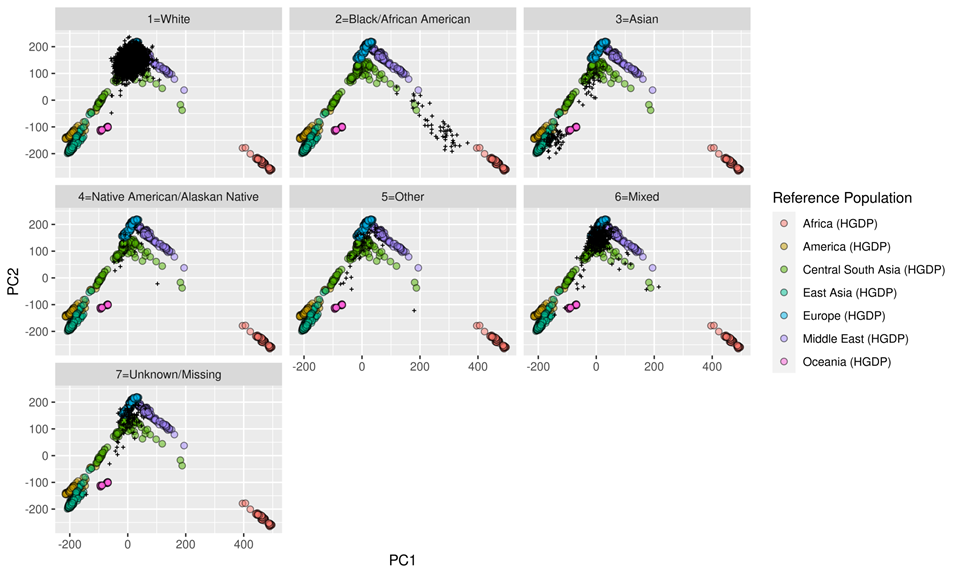

Supplement: Supplementary file 5 — Supplemental Figure 2 – Leading principal components from TRACE with RIGHT participants projected onto reference samples from HGDP [file 41397_2022_265_MOESM5_ESM.png]

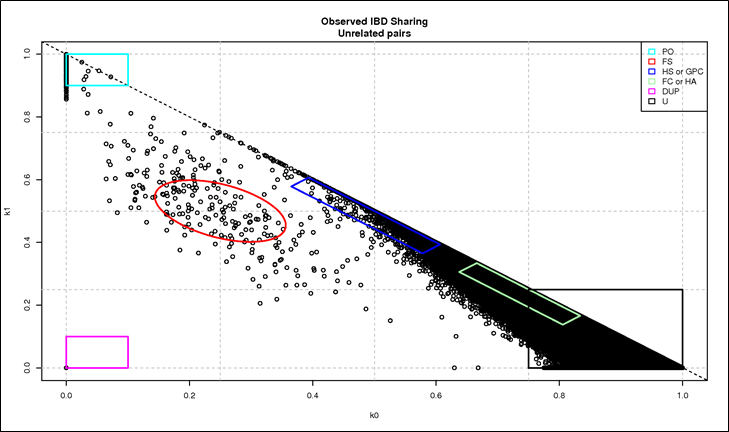

Supplement: Supplementary file 6 — Supplemental Figure 3 – IBD estimates for relatedness checks in RIGHT using PREST [file 41397_2022_265_MOESM6_ESM.png]

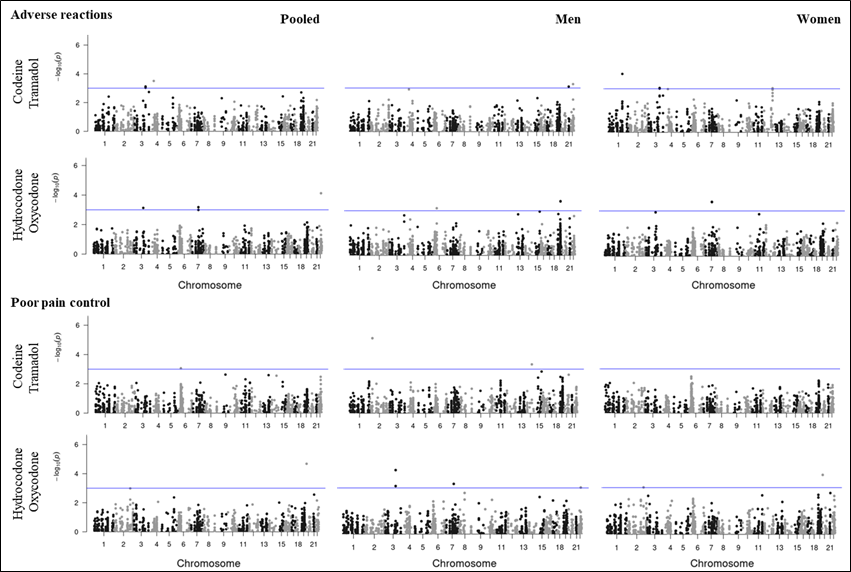

Supplement: Supplementary file 7 — Supplemental Figure 4 – Manhattan plot of PGx variant associations with opioid response by sex [file 41397_2022_265_MOESM7_ESM.png]
